# A contemporary approach to a young female patient with Loeys-Dietz syndrome and an uncomplicated type B aortic dissection: a case report

**DOI:** 10.1186/s13019-020-01274-0

**Published:** 2020-08-31

**Authors:** Petko Prodanov, Hana Linkova, Robert Petr, Richard Fojt, Zuzana Motovska, Jiri Knot, Filip Rohac, Boris Koznar, Mariwan Majid, Petr Widimsky, Petr Kacer

**Affiliations:** 1 Department of Cardiac surgery, Faculty Hospital Královské Vinohrady, Srobarova 50, 10034 Praha, Czech Republic; 2 3rd Department of Internal Medicine – Cardiology, Faculty Hospital Královské Vinohrady, Srobarova 50, 10034 Praha, Czech Republic

**Keywords:** Acute aortic syndrome, Aortic disease, Aortic dissection, Connective tissue disorder

## Abstract

**Background:**

Aortic dissection is a relatively uncommon, but often catastrophic disease that requires early and accurate diagnosis. It often presents in patients with congenital connective tissue disorders. The current aortic surgical techniques are related with serious early and late complications. This case report emphasizes the importance of early diagnosis of aortic root dilatation and the risk of dissection, especially in patients with congenital connective tissue disorders. We present an alternative, contemporary and multidisciplinary approach based on the present state of knowledge.

**Case presentation:**

We present a rare case of a young female patient with Loeys-Dietz syndrome who was admitted with an uncomplicated aortic dissection (Stanford type B / DeBakey type III) and a dilated aortic root. After a period of close surveillance and extensive vascular imaging, thoracic endovascular aortic repair was deemed to be technically not possible. Medical treatment was optimized and our patient successfully underwent a personalised external aortic root support procedure (PEARS) as a contemporary alternative to existing aortic root surgical techniques.

**Conclusions:**

This case highlights the importance of interdisciplinary approach, close follow-up and multimodality imaging. The decision to intervene in a chronic type B aortic dissection is still challenging and should be made in experienced centers by an interdisciplinary team. However, if an acute complication occurs, thoracic endovascular aortic repair TEVAR is the method of choice. In all cases optimal medical treatment is important. There is increasing evidence that personalized external aortic root support procedure PEARS is effective in stabilizing the aortic root and preventing its dilatation and dissection not only in patients with Marfan syndrome, but also in other cases of aortic root dilation of other etiologies. Moreover, many publications have reported the additional benefit of reduction or even eradication of aortic regurgitation by improving coaptation of the aortic valve leaflets in dilated aortas.

## Background

Connective tissue disorders such as Marfan syndrome, Loeys–Dietz syndrome, Ehler–Danlos syndrome, inflammatory diseases of the aorta, Turner syndrome and bicuspid aortic valves are associated with medial degeneration and early onset of aortic dissection. Younger people are more commonly affected and pregnancy poses a risk of catastrophic complications. Total root replacement, also known as the Bentall operation, and valve sparing root replacement (VSRR) surgery are associated with long recovery periods and severe complications. There is increasing evidence that the personalized external aortic root support (PEARS) procedure is effective in stabilizing the aortic root and preventing its dilatation and dissection not only patients with Marfan syndrome, but also for other causes of aortic root dilation.

**Fig. 1:**
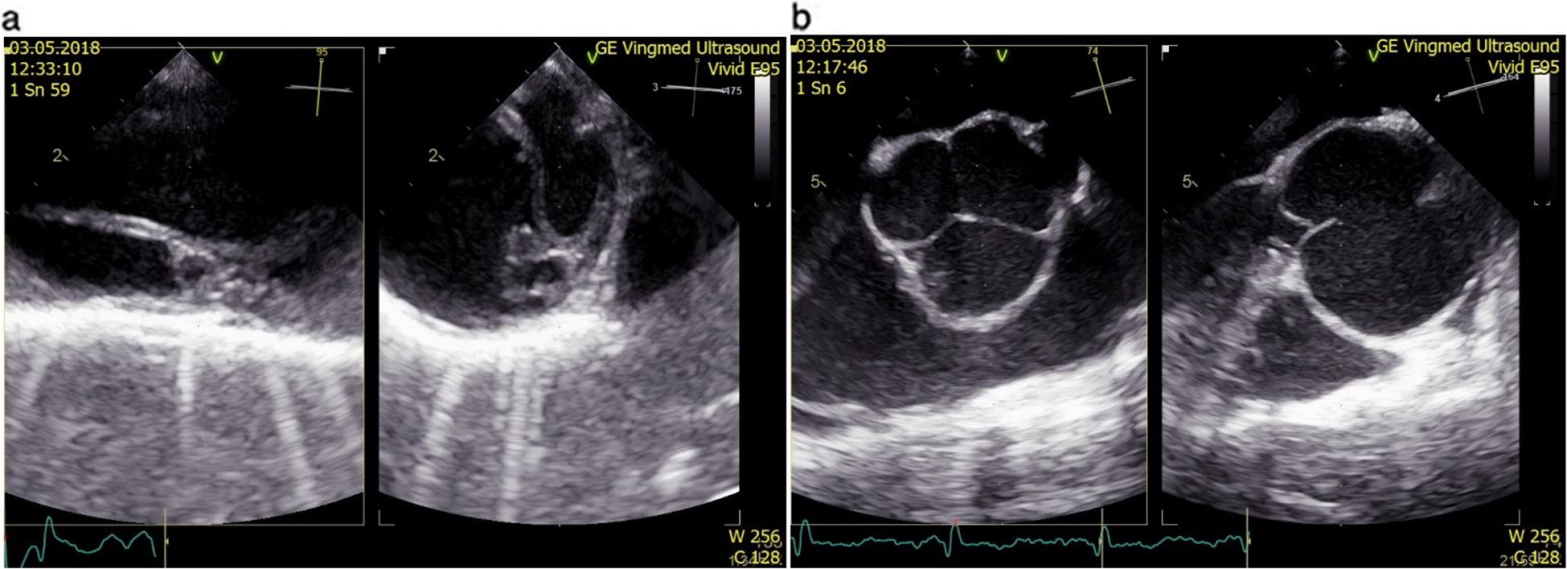
**a** Transesophageal echocardiography showing an intimal flap through the whole length of the descending aorta with a small mobile echogenic structure in the proximal part of the false lumen **b** Dilated aortic root and tricuspid aortic valve

**Fig. 2:**
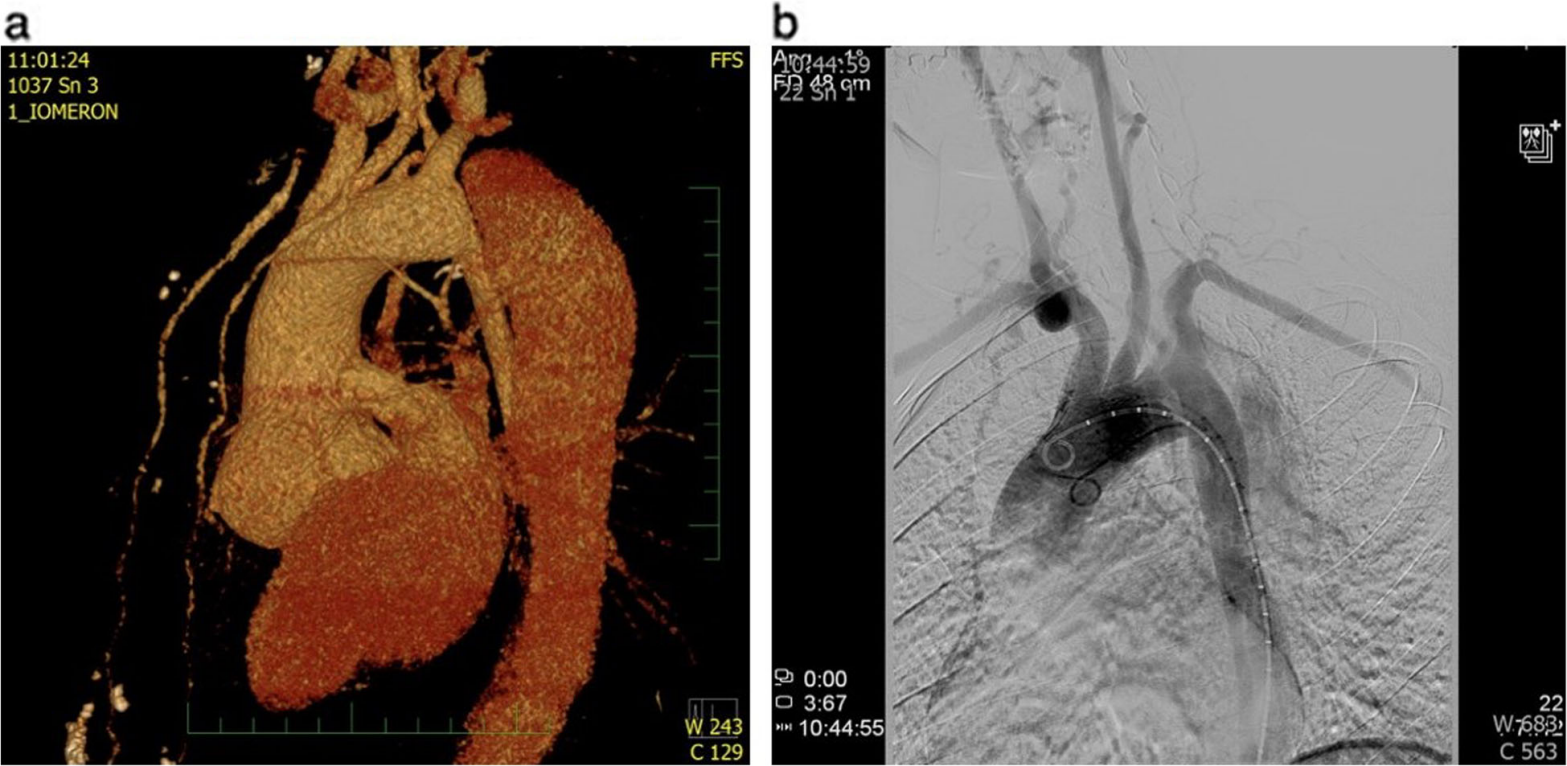
**a** A CT angiography reconstruction showing the location of the proximal entry tear located just after the origin of the subclavian artery. The distal re-entry tear was located at the level of the visceral arteries. **b** Digital subtraction angiography confirming the type B aortic dissection

**Fig. 3:**
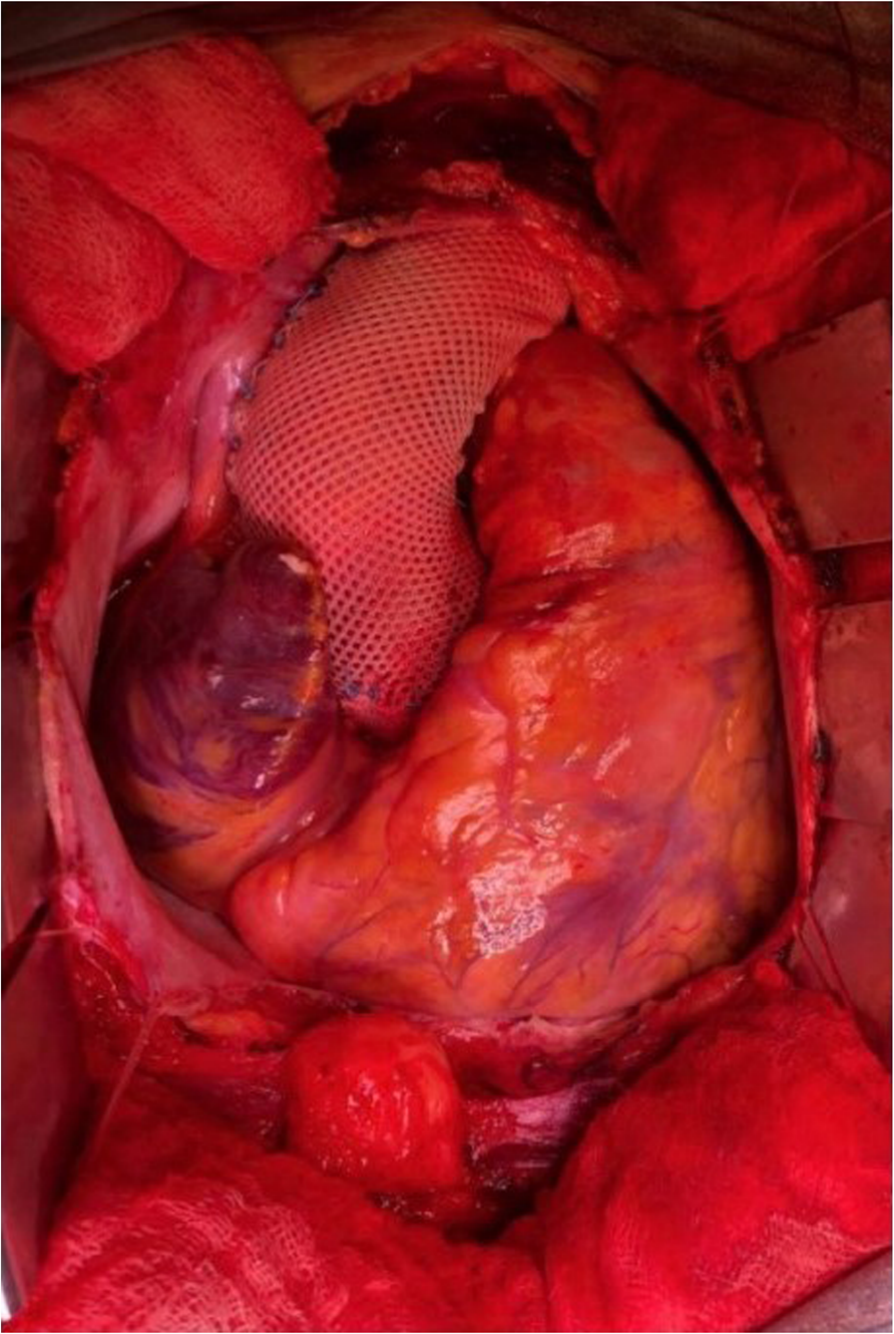
The custom-made external support is a pliable macroporous textile mesh designed to exactly match the patient’s aortic root without disrupting the coronary arteries. The operation can be performed without the use of extracorporeal circulation and heparinization

**Fig. 4:**
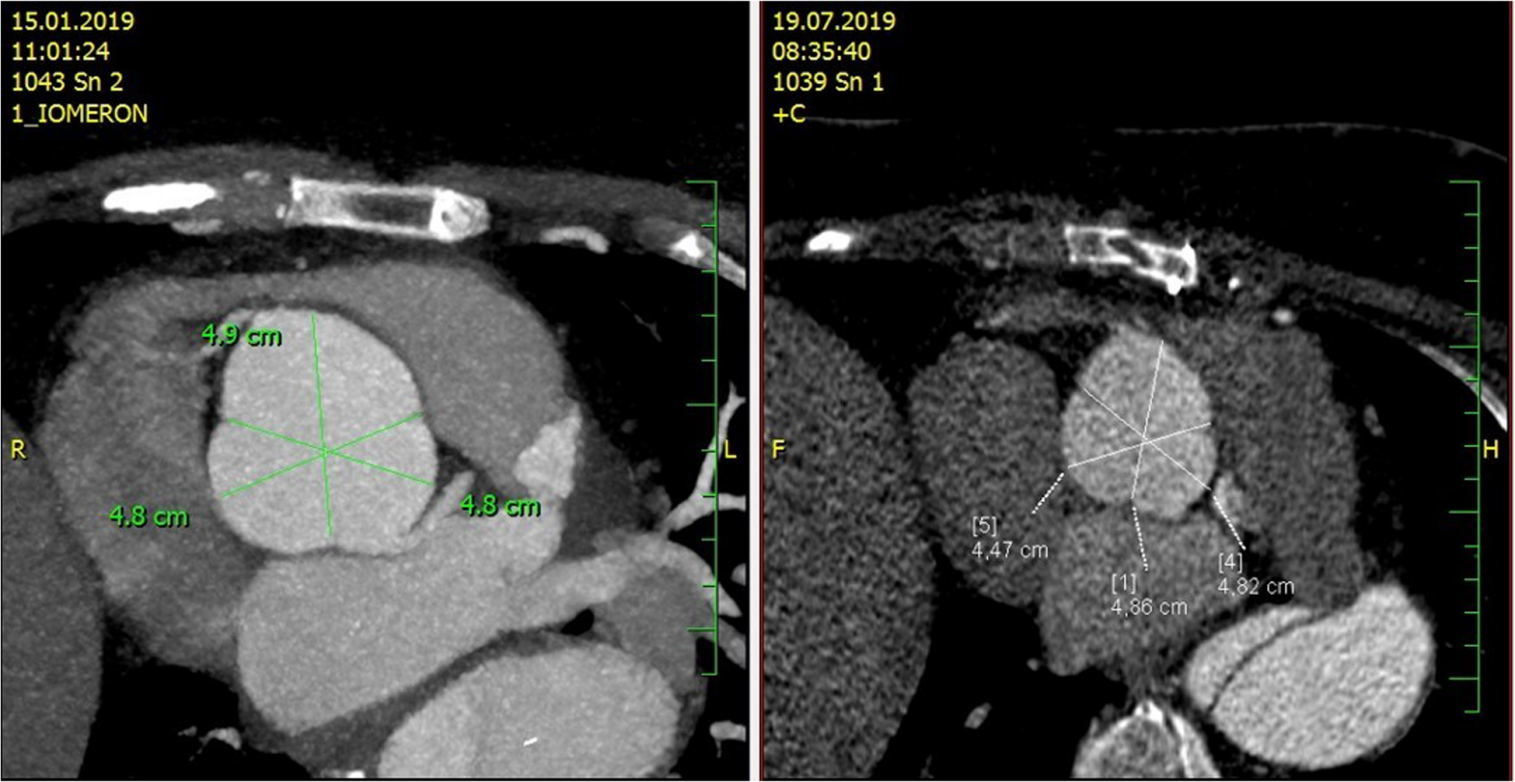
CT AG before PEARS and at 14 months after PEARS procedure

According to the European Society for Cardiology guidelines of aortic disease from 2014 in the absence of organ malperfusion or signs of early progression of Type B aortic dissection patients can be safely stabilized under medical therapy used to control pain and blood pressure. Beta blockers should be administered to all patients with Marfan syndrome and aortic aneurysms unless contraindicated. Angiotensin-converting enzyme inhibitors or angiotensin receptor blockers are reasonable choices for reducing blood pressure to the lowest point patients can tolerate without adverse effects [1]. If an acute complication occurs thoracic endovascular aneurysm repair (TEVAR) is the method of choice. Nowadays surgery is rare in cases of complicated Type B aortic dissection and is related with a much higher risk of complications and in-hospital mortality rate of 25–50% [2]. When there is no proximal landing zone for TEVAR procedure, the “frozen elephant trunk” technique might be considered. The decision to intervene in a chronic type B aortic dissection is still challenging and should be made in experienced centers.

## Case presentation

A 39-years-old female, mother of 3 children, with a history of intracerebral hemorrhage at the age of one and with a family history of sudden death in a second degree relative, presented in May 2018 at the emergency department of the University Hospital Královské Vinohrady in Prague after an episode of chest pain and shortness of breath that occurred during a funeral ceremony. At the time of admission, the physical examination revealed no abnormal findings, her blood pressure was 155/80 mmHg, her height was 185 cm and weight was 95 kg. She was on standard antidepressant therapy and flavonoids.

At the time of admission, the electrocardiogram was normal, D-dimers were elevated to 4170 μg/l and high-sensitive troponin T was negative. The CT computed tomography (CT) angiography revealed a dilated aortic root and a Stanford type B aortic dissection. The left vertebral artery originated atypically from the true lumen of aortic arch as a third branch just before the origin of left subclavian artery. The right vertebral artery was hypoplastic. The left renal and accessory renal artery flow originated from the false lumen and the dissection extended to the origin of the left common iliac artery. Transesophageal echocardiography showed an intimal flap extending through the whole length of the descending aorta with a small mobile echogenic structure in the proximal part of the false lumen. The aortic root was dilated to 51 × 54 mm and the aortic valve was tricuspid with an insignificant central regurgitation jet. The left ventricular function was normal, without regional wall motion abnormalities. Mild regurgitation of the mitral and tricuspid valves without signs of mitral valve prolapse was noted (Fig. 1a, b). Digital subtraction angiography was also performed and confirmed the type B aortic dissection. It also revealed that compression of the true lumen is not as significant as the CT angiography showed before. The proximal entry tear was located immediately distal to the origin of left subclavian artery and the distal re-entry was located at the level of the visceral arteries (Fig. 2a, b).

At the time of admission, the approach was conservative. Considering the stable clinical condition of the patient, with no recurrent pain, no signs of malperfusion or rupture, acute intervention was not indicated. Antihypertensive medication was administered and up-titrated until effective control of blood pressure was achieved. The CT angiography was repeated 15 days after admission. The results showed progression of the size of descending aorta from 35 mm to 40 mm with compression of the true lumen in the thoracoabdominal region, but with no clinical or laboratory correlation or symptoms. The hospitalization duration was 32 days and no complications occurred. The patient was discharged on a combination of antihypertensive drugs - carvedilol, perindopril, amlodipine and urapidil. After 3 months CT angiography was performed again. The results showed slight uncomplicated progression of the descending aorta to 44 mm, with no further progression of the aortic root size.

Genetic studies revealed autosomal dominant connective tissue disorder with heterozygous mutations c.605C > T in gene TGFBR1 which is associated with Loeys-Dietz syndrome. According to the current guidelines for aortic diseases from 2014 management should be tailored in reference to extensive vascular imaging at baseline and family history of vascular events [1].

Further therapeutic approach was considered by the Heart team in collaboration with angiologists and vascular surgeons. As a contemporary less invasive alternative to existing ascending aorta procedures, our patient underwent a personalized external aortic root support procedure (PEARS). The procedure was performed successfully without the use of cardiopulmonary bypass. After 8 days of hospitalization the patient was discharged (Fig. 3).

As for the aortic dissection, the Heart team in collaboration with angiologists and vascular surgeons recommended a conservative approach with close surveillance of the patient. Thoracic endovascular aortic repair or open surgery would only be considered if any complication occurred or if the size of descending aorta showed rapid further progression. The CT angiography performed 14 months after the first medical contact demonstrates excellent effect of the PEARS procedure with stability of the ascending aortic dimensions and morphology and does not show further progression of the size of the descending aorta (Fig. 4).

Meanwhile, all three children of our patient underwent a cardiologic examination. In all of them the ascending aorta was dilated and genetic studies confirmed gene mutation related with Loeys-Dietz syndrome. The oldest child underwent aortic root operation; the others are under close surveillance.

## Discussion

There is little data comparing TEVAR with optimal medical therapy in patients with uncomplicated Type B aortic dissection. The Investigation of Stent Grafts in Patients with Type B Aortic Dissection (INSTEAD) trial showed that TEVAR has no clinical benefit over medical therapy [3]. On the other hand an extended follow-up of this study (INSTEAD-XL) and observations from the IRAD registry show that aorta-related mortality and disease progression were significantly lower after 5 years in TEVAR patients compared with those receiving medical therapy only [4, 5].

The Acute Dissection Stent Grafting or Best Medical Treatment (ADSORB) results published in 2017 identified the number of vessels originating from the false lumen as an independent predictor of false lumen growth in uncomplicated type B aortic dissection. The study also described the clear benefit of stent graft placement on aortic remodeling and increased rates of false lumen thrombosis in the intervention group compared to optimal medical treatment [6].

In our case, regarding the location of proximal intimal tear of the AD, obliteration by implantation of a membrane-covered stent-graft seems to be technically impossible because of insufficient proximal zone of anchoring and risk of iatrogenic occlusion of the left subclavian or/and left vertebral artery. However, if an acute complication occurs, TEVAR is the treatment of choice [7]. Despite the surgical results improvement over past decades, they remain suboptimal, with in-hospital mortality ranging from 25 to 50% [2].

As an alternative procedure to the *existing ascending aorta surgery practice,* there is increasing evidence that PEARS is effective in stabilizing the aortic root and preventing its dilatation, secondary aortic regurgitation and aortic dissection in patients with connective tissue disorder [8]. However, there is further evidence that it is also applicable to aortic root aneurysms of other etiologies. It is the morphology of the aortic root aneurysm and the risk of dissection and rupture that are more important in the present state of knowledge, not the genotype [9]. The custom-made external support is a pliable textile macroporous mesh designed to exactly match the patient’s aortic root. It is prefabricated by using 3-D printed plastic former based on a 3-D model of MRI or CT images, which provides the shape. In contrast with aortic wrapping, the microporous mesh of PEARS is intimately in contact with the aorta due to its personalized design and does not show any signs of tissue necrosis. Histological examination shows that the microporous mesh fully incorporates with collagen fibers and allows recovery of the microstructure of the media [10]. However the due to the nature of the PEARS it can only be performed as an elective procedure while wrapping can be undertaken in an emergency setting. The PEARS procedure can be performed without the use of extracorporeal circulation and heparinization. Coronary arteries stay intact and since the vessel and the valve remain native there is no need for the patient to take anticoagulant medication.

According to a project update, published by Golesworthy [11], as of March 2020, 317 patients have been treated with personalized external aortic root support surgery. The most common underlying condition of these patients was Marfan syndrome (*n* = 177). Other pathologies included bicuspid aortic valve (*n* = 24), Loeys-Dietz syndrome (*n* = 18), idiopathic aortic dilation (*n* = 19), transposition of the great arteries repaired by a switch operation (*n* = 3), aortic valve disease treated by the Ross procedure (*n* = 28), ACTA2 mutation (*n* = 3), tetralogy of Fallot (*n* = 1) and 38 cases not yet reported. The follow up of patients demonstrates stability of the aortic dimensions and morphology over a significant time period of 5 to 15 years, with perioperative mortality rate of 0.34%. At least four female Marfan patients after PEARS procedure subsequently had 5 uneventful pregnancies and deliveries without evidence of further aortic dilatation.

## Conclusion

The decision to intervene in a chronic aortic dissection type B is still challenging and should be made only in experienced centers. However, if an acute complication occurs TEVAR is the method of choice [7].

As an alternative procedure to TEVAR, PEARS is safe and effective in stabilizing the aortic root and preventing its dilatation and dissection in carefully selected patients with connective tissue disorders [8]. The future results from prospective cohort studies comparing PEARS with total root replacement and aortic valve sparing root replacement will be important to understand if patients receiving PEARS have had an unnecessary intervention and with a higher risk of complications. However, according to the NHS Clinical Commissioning Policy the current inclusion criteria for PEARS as surgical management of enlarged aortic root in adults appears to be safe and effective as the results for valve related deaths and complication rates are low.

## Data Availability

Not applicable.

## Abbreviations

PEARS: Personalised external aortic root support procedure
TEVAR: Thoracic endovascular aortic repair
CT: Computed tomography
